# Probiotic *Lactobacillus plantarum* LP28 and *Saccharomyces cerevisiae* improve the bioactive content and quality of fruit‐based rice beverage

**DOI:** 10.1002/fsn3.4462

**Published:** 2024-10-06

**Authors:** Afusat Yinka Aregbe, Turkson Antwi Boasiako, YuQing Xiong, Md. Hafizur Rahman, Yongkun Ma

**Affiliations:** ^1^ School of Food and Biological Engineering Jiangsu University Zhenjiang China; ^2^ Department of Quality Control & Safety Management, Faculty of Food Sciences & Safety Khulna Agricultural University Khulna Bangladesh

**Keywords:** antioxidant properties, E‐nose, phytochemicals, rice beverage, volatile compounds

## Abstract

The increasing demand for plant‐based beverages with improved functional and sensory qualities has guided this study, which examines the bioactive content, functional, and sensory properties of a rice, apple pomace, and sea buckthorn beverage (RASB) fermented with probiotic *Lactobacillus plantarum* and *Saccharomyces cerevisiae*. We found out that total polyphenol content (TPC), total flavonoid content (TFC), and β‐carotene were significantly higher in samples with *Saccharomyces cerevisiae*, particularly in coculture samples. These samples also exhibited elevated alcohol by volume (ABV). Monoculture samples showed increased total flavonol content (TFLC), total anthocyanin content (TAC), and proanthocyanidin. The RASB‐LP sample, containing only *Lactobacillus plantarum*, revealed the highest antioxidant properties, evidenced by DPPH (94.13 ± 0.05%) and ABTS (97.69 ± 0.09%) assays. Interestingly, 3‐methylbutyl 3‐methylbutanoate, abundant in the unfermented control, was hydrolyzed to 3‐methyl‐1‐butanol in fermented samples, especially those containing *Saccharomyces cerevisiae*. Sensory evaluation evidenced that RASB‐LP scored highest for aroma and overall acceptability. FTIR analysis also indicated changes in functional groups of RASB samples. Together, our findings suggest that a novel probiotic cereal beverage with enhanced quality can be developed through the addition of fruit and fruit pomace, coupled with fermentation using *Lactobacillus plantarum* LP28 and *Saccharomyces cerevisiae*.

## Graphical abstract

The study examined a rice, apple pomace, and sea buckthorn beverage (RASB) fermented with *Lactobacillus plantarum* and *Saccharomyces cerevisiae*. Coculture samples had significantly higher total polyphenol content (TPC), total flavonoid content (TFC), and β‐carotene, while RASB‐LP showed the highest antioxidant properties and best sensory scores. FTIR analysis indicated functional group changes. Our findings suggest that high‐quality probiotic cereal beverages can be developed through fruit pomace addition and fermentation with *Lactobacillus plantarum* LP28 and *Saccharomyces cerevisiae*.

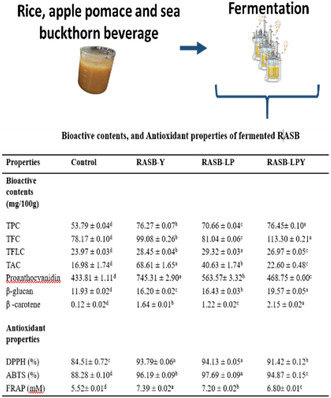

## 1 INTRODUCTION

Plant‐based beverages (PBBs) are gaining popularity for their health benefits and suitability for lactose‐intolerant individuals. However, they face challenges due to the availability and variations in nutrients and bioactive ingredients in their raw materials (Patra et al., 2023), which necessitates the exploration of indigenous sources. Rice, a global staple and a key ingredient in various breakfast products, including cereal‐based beverages particularly in Asia, often lacks essential nutrients, especially in its commonly consumed white variety (Giri et al., 2018). This deficiency, coupled with the widespread consumption and demand for higher nutritional quality, highlights the need for enhanced nutritional and sensory profiles. Supplementation with nutrient‐ and antioxidant‐rich fruits or fruit pomace can improve the taste and nutritional value. Moreover, employing fermentation technology can further enhance these properties in rice‐based beverages.

Sea buckthorn (*Hippophae rhamnoides* L.), renowned for its nutritional and medicinal values, is native to China. Apple pomace, a nutrient‐ and phytochemical‐rich byproduct of apple juice production, generates a significant environmental concern in China, particularly in the Xinjiang province, due to waste management challenges. Integrating apple pomace into a new rice beverage could mitigate this issue. Meanwhile, the bioactive components found in both sea buckthorn and apple pomace have been reported to offer various health benefits, including free radical scavenging abilities, and the potential to lower blood pressure, cholesterol, and sugar levels (Ge et al., 2022).

Fermentation is vital in the production of cereal‐based products, significantly enhancing their organoleptic, functional, and nutritional qualities, and extending shelf life. Lactic acid bacteria (LAB), especially *Lactobacillus plantarum*, along with yeasts such as *Saccharomyces cerevisiae*, have been utilized to improve the quality of fermented foods, including rice‐based beverages (Giri et al., 2018; Mu et al., 2023). These microorganisms secrete enzymes like glucosidase, amylase, cellulase, and esterase, aiding in breaking down cell walls and converting complex organic compounds into simpler forms (Malini et al., 2024), thus increasing bioactive components and functionality. Giri et al. (2018) observed enhanced antioxidant properties and aroma profiles in *Lactobacillus plantarum* L7 fermented rice beverages, while Mu et al. (2023) reported improved aroma in *Saccharomyces cerevisiae* fermented Chinese rice wine.

Despite numerous studies on fermenting cereal beverages with *Lactobacillus plantarum* and *Saccharomyces cerevisiae*, research on fermenting these beverages with fruit and pomace additions is limited. Yet, incorporating fruit pomace into the diet not only contributes to healthier food choices but also aligns with efforts to create a more sustainable, cost‐effective, and less wasteful food system. Therefore, our study aims to evaluate the bioactive content, and functional and sensory properties of a rice, apple pomace, and sea buckthorn beverage fermented with *Lactobacillus plantarum* and *Saccharomyces cerevisiae*.

## 2 MATERIALS AND METHODS

### 2.1 Raw materials, reagents, and microbial starter

Rice was purchased from a supermarket in Zhenjiang, Jiangsu Province, China. Sea buckthorn pulp and apple pomace were sourced from Xinjiang Province, China. *Lactobacillus plantarum* LP28 was acquired from Synbio Tech Inc (Kaohsiung City, Taiwan) while active dry wine yeast (*Saccharomyces cerevisiae*) was obtained from Angel Yeast Co., Ltd (Hubei, China). All analytical grade reagents were acquired from Sinopharm Chemical Reagent (Shanghai, China).

### 2.2 Inoculum preparation

A total of 0.01 g of *Lactobacillus plantarum* was subjected to activation in 150 mL of MRS broth (De Man, Rogosa, and Sharpe) for 24 h at 37°C. Successively, the microorganism underwent subculturing cycles twice and centrifugation at 3000 × *g* for 10 min, at 4°C. The microbial cells were washed using 0.1% NaCl solution and then resuspended in sterile distilled water. Similarly, 0.01 g of yeast was activated with 50‐mL warm sterile distilled water containing 0.1 g sucrose. Inoculum concentration for LAB and yeast was established through the plate count method using MRS agar and yeast extract peptone dextrose (YPD) agar, resulting in quantification of 7.83 Log CFU for *L. plantarum* and 6.92 Log CFU for *Saccharomyces cerevisiae*.

### 2.3 Beverage preparation and fermentation

Rice (50%), apple pomace (25%), and sea buckthorn pulp (25%) were blended with sterile distilled water (1:10 w/v) using a blender (Jujike, Foshan Kelaiya Electrical Appliance Co. Ltd., Foshan City, China). This mixture was sieved and gelatinized at 70°C for 10 min. The initial°Brix of 4 was adjusted to 11°Brix with sucrose and the pH adjusted to 5.5. The mixture was inoculated with 1% (v/v) of each inoculum and the samples were fermented for 48 h at 30°C. An unfermented sample was used as a control.

### 2.4 Physicochemical properties, microbial, and color analysis

#### 2.4.1 pH, total soluble solids (TSS), total titratable acidity (TTA), and alcohol by volume (ABV)

The pH was measured using a digital pH meter (LIDA Instrument PHS‐3C Precision pH/mV meter). The TSS was determined using an Atago digital refractometer (Japan) and expressed as°Brix. TTA was carried out according to the method described by Liu et al. (2023). A 1 mL of the sample was mixed with 50 mL of distilled water. Thereafter, 1–2 drops of phenolphthalein were added to the mixture. TTA was determined by titration with 0.1 M NaOH and was calculated using the conversion coefficient of lactic acid. The TTA was calculated as follows:

(1) $$\text{Titratable acidity}\left(\frac{g}{L}\right)=\frac{C\times \left(V_{1}-V_{2}\right)\times 0.090\times F}{V}\times 1000$$

where C (mol/L) is the concentration of 0.1 NaOH standard titration solution; *V*_1_ (mL) is the final volume of NaOH used for titration; *V*_2_ (mL) is the initial volume of NaOH before titration; the conversion coefficient of lactic acid is 0.090; *F* is the dilution factor; *V* (mL) is the volume of sample.

Alcohol by volume (ABV) was calculated using the following formula:

(2) $$\mathrm{ABV}\;\left(\%\right)=\left(\mathrm{SPG}1-\mathrm{SPG}2\right)\times 131.25$$

where SPG1 is initial specific gravity, SPG2 is the final specific gravity.

#### 2.4.2 Microbial analysis

Microbiological analysis was performed using the method described by Kwaw et al. (2017).

### 2.5 Aroma determination

#### 2.5.1 Electronic nose (E‐nose) and sensory evaluation

E‐nose analysis was conducted using a PEN3.5 e‐nose (Airsense Analytics Inc., Schwerin, Germany), equipped with 10 metal‐oxide semiconductor sensors (W1C, W5S, W3C, W6S, W5C, W1S, W1W, W2S, W2W, and W3S) following the procedure established by Boasiako et al. (2024). Each sample (5 mL) was placed in vials fitted with a Teflon/silicon septum in the screw lid. The analysis program included a flush time of 180 s, an automatic zero adjustment spanning 10 s, internal and inlet flow rates set at 600 mL/min, and a measurement time of 150 s.

Sensory evaluation was conducted according to the method previously reported by Kwaw, Ma, Tchabo, Sackle, et al. (2018), with slight modifications. The assessment was performed using 20 untrained staff of Food and Biological Engineering, ranging in age from 25 to 45 years. Informed consent was obtained from the participant before the sensory test was carried out. The samples were evaluated based on color, aroma, taste, flavor, mouthfeel, and overall acceptability using a 9‐point hedonic scale (1 = extremely dislike; 2 = very much dislike; 3 = dislike; 4 = slightly dislike; 5 neither like nor dislike; 6 = slightly like; 7 = like; 8 = like very much; 9 like extremely).

#### 2.5.2 Volatile compounds analysis

The analysis of volatile components was performed using the HS‐SPME system (Agilent Technologies, Santa Clara, CA USA) as described by Boasiako et al. (2024) with slight modification. Ten microliters of 2‐octanol (800 μg/L) was used as an internal standard in a 5‐mL sample with 1.5 g NaCl, equilibrated at 40°C for 20 min. A DVB/CAR/PDMS fiber (50/30 μm, Fisher Scientific) was then exposed to the sample's headspace for 30 min, agitated at 2.5 Hz. Postexposure, the fiber was desorbed at 250°C for 4 min in the GC–MS's injection port using an Rtx‐WAX column (Restek, USA). Analysis conditions included a spitless injection, helium as the carrier gas at 1 mL/min, with a temperature program from 50°C to 240°C. The MS scan from 33 to 350 amu, with compounds identified using the NISIT 17 library. Volatile compounds with a matching degree of >85% were considered and semiquantification was done using 2‐octanol as an internal standard following the equation below:

(3) $$\mathrm{VC}\left(\frac{\mathrm{ng}}{g}\right)=\frac{\text{Peak area ratio}\times 10\;\mathrm{\mu L}\;\left(\mathrm{ISD}\right)\times 0.8\left(\frac{\mathrm{ng}}{g}\right)\;\left(\mathrm{ISD}\right)}{\text{equivalent mass of volume used}\;}$$

### 2.6 Fourier‐transform infrared spectroscopy (FTIR)

The FTIR analysis was conducted following the method outlined by Papadopoulou et al. (2021). The infrared spectrum was recorded over a range of 4000–600 cm^−1^, using a FT‐IR spectrometer (NICOLET iS50 FT‐IR spectrometer, Thermo Scientific).

### 2.7 Phytochemical analysis

The total polyphenol content (TPC) of RASB was determined using the Folin–Ciocalteu method, while the total anthocyanin content (TAC) was determined using a pH differential method as described by Kwaw, Ma, Tchabo, Apaliya, et al. (2018). The total flavonoid content (TFC) was analyzed in accordance with Kwaw, Ma, Tchabo, Apaliya, et al. (2018). Meanwhile, the method by Kumaran and Karunakaran (2007) was followed for the determination of total flavonol content (TFLC). The absorbance was read using an ultraviolet spectrophotometer (UV‐1600, Beijing Rayleigh analytical instrument, Beijing, China). The proanthocyanidin content of RASB was determined according to a previously reported method by Boasiako et al. (2024) while the β‐glucan and β‐carotene content were quantified as described by Anderson (1990) and Hasan et al. (2022), respectively.

### 2.8 Antioxidant properties

#### 2.8.1 DPPH radical scavenging activity

The DPPH radical scavenging activity DPPH^·^ – SA (2, 2‐diphenyl‐1‐picrylhydrazyl) analysis was evaluated according to the procedure described by Li et al. (2022) with slight modification. Briefly, 4.2 mL of 0.1 mM DPPH solution was added to 0.12 mL of the sample (diluted 1:15). After vortexing, the mixture was incubated for 30 min at 25°C in the dark. The absorbance was measured using an ultraviolet spectrophotometer (UV‐1600). The control was RASB without DPPH. The result was expressed as the percentage of DPPH^·^‐SA using Equation 3 as follows:

(4) $$\%{\text{DPPH}}^{\cdot }-\mathrm{SA}=\left(\frac{A_{\text{control}}-A_{\text{sample}}}{A_{\text{sample}}}\right)\times 100$$

#### 2.8.2 ABTS radical cation scavenging activity (an electron transfer‐based assay)

The ABTS (2,2′‐azino‐bis (3‐ethylbenzothiazoline‐6‐sulfonic acid)) radical cation scavenging activity was performed in line with the procedure described by Kwaw, Ma, Tchabo, Apaliya, et al. (2018).

#### 2.8.3 Ferric reducing antioxidant power (FRAP) capacity

FRAP assay was performed according to the procedures outlined by Feng et al. (2019) with slight modifications. Briefly, 0.2 mL of the beverage sample was mixed with 3.8 mL of FRAP reagent (a mixture of 10 parts 300 mM sodium acetate buffer (pH 3.6), 1 part 10 mM TPTZ (2, 4, 6‐tripyridyl‐s‐triazine) in 40 mM HCl, and 1 part 20 mM FeCl_3_·6H_2_O). The resulting solution was incubated for 30 min at 37°C. Afterward, the absorbance was measured at 593 nm. The results were reported in mM FeSO_4_.

### 2.9 Statistical analysis

Treatments were performed in triplicate and results were presented as mean ± standard deviation. ANOVA was performed using SPSS version 26 while Pearson correlation analysis was performed using OriginLab 2021. Duncan's test was used to compute a significant difference at *p* < .05.

## 3 RESULTS AND DISCUSSION

### 3.1 Changes in pH, total titratable acidity (TTA), total soluble solids (TSS), and microbial counts during fermentation

Table 1 displays the results of pH, TTA, TSS, and microbial counts throughout the fermentation period. pH consistently decreased during fermentation, accompanied by a concomitant increase in TTA. However, no significant difference was observed in TTA at 24 h. The decrease in pH and increase in TTA can be attributed to the production of organic acids resulting from the metabolic activities of the fermenting microorganisms (Li et al., 2021). Simultaneously, a marked TSS decrease occurred in the samples containing *Saccharomyces cerevisiae* (RASB‐Y, RASB containing *Saccharomyces cerevisiae*, and RASB‐LPY, RASB containing *L. plantarum* LP28 and *Saccharomyces cerevisiae*) exhibited lower TSS values, with the lowest value recorded in RASB‐Y at 24 h (10°Brix) and 48 h (9°Brix). Regarding microbial counts, the LAB count was significantly higher in the monoculture RASB‐LP (RASB, containing *L. plantarum* LP28) compared to the bi‐culture sample, particularly at 48 h. The LAB count increased from 10.55 log CFU/mL (24 h) to 12.25 log CFU/mL (48 h), while a slight decrease was observed in the coculture sample, as presented in Table 1. The decrease in LAB count in the coculture sample might be attributed to competition for nutrients. A significant difference was observed in the yeast counts. Although the monoculture sample had the highest value at 24 and 48 h, a remarkable increase occurred in the bi‐culture sample from 24 h (7.46 log CFU/mL) to 48 h (8.25 log CFU/mL). The increase observed in RASB‐LPY might be due to the low pH recorded in this sample, suggesting a synergy between *L. plantarum* and *Saccharomyces cerevisiae*. Freire et al. (2017) had earlier reported that pH reduction by LAB could favor the growth of yeast.

**TABLE 1:** Changes in pH, total titratable acidity (TTA), total soluble solids (TSS), and microbial counts during fermentation.

Sample	pH	
	24 h	48 h
RASB‐Y	4.65 ± 0.01^a^	3.70 ± 0.01^a^
RASB‐LP	3.77 ± 0.01^b^	3.12 ± 0.01^b^
RASB‐LPY	3.56 ± 0.01^c^	3.01 ± 0.01^c^
	**TTA (g/L lactic acid)**	
RASB‐Y	22.95 ± 0.00^a^	45.90 ± 0.00^b^
RASB‐LP	30.60 ± 6.63^a^	68.85 ± 0.00^a^
RASB‐LPY	22.95 ± 0.00^a^	53.55 ± 13.25^b^
	**TSS (°Brix)**	
RASB‐Y	10.00 ± 0.00^b^	9.00 ± 0.00^a^
RASB‐LP	10.83 ± 0.29^a^	9.50 ± 0.50^a^
RASB‐LPY	10.17 ± 0.29^b^	9.17 ± 0.29^a^
	**LAB (log**_**10**_ **Cfu/mL)**	
RASB‐Y	ND	ND
RASB‐LP	10.55 ± 0.02^a^	12.25 ± 0.04^a^
RASB‐LPY	10.42 ± 0.04^b^	10.32 ± 0.05^b^
	**Yeast (log**_**10**_ **Cfu/mL)**	
RASB‐Y	8.34 ± 0.05^a^	8.51 ± 0.04^a^
RASB‐LP	ND	ND
RASB‐LPY	7.46 ± 0.03^b^	8.25 ± 0.08^b^

*Note*: RASB‐Y represents RASB inoculated with *Saccharomyces cerevisiae*, RASB‐LP represents RASB inoculated with *Lactobacillus plantarum* LP28, RASB‐LPY represents RASB inoculated with *Lactobacillus plantarum* LP28 and *Saccharomyces cerevisiae*. RASB represents rice, apple pomace, and sea buckthorn beverage. Means with different letters are significantly different (*p* < .05).

### 3.2 Alcohol by volume (ABV)

The ABV was higher in samples containing *Saccharomyces cerevisiae* (RASB‐Y and RASB‐LPY) while RASB‐LP recorded the lowest value 0.73 ± 0.15% as shown in Table 2. The high ABV in RASB‐Y and RASB‐LPY can be attributed to the presence of *Saccharomyces cerevisiae* and the ability to convert sugar into alcohol. The low TSS values recorded in these samples could justify the increase in ABV. The ABV (alcohol by volume) range obtained in this study, 0.73%–1.09%, falls within the category of low‐alcohol beverages, which is lower than the 1.2% reported by Patra et al. (2023) for alcoholic beverages.

**TABLE 2:** Bioactive contents, alcohol by volume (ABV) and antioxidant properties of fermented RASB.

Properties	Control	RASB‐Y	RASB‐LP	RASB‐LPY
Bioactive contents (mg/100 g)				
TPC	53.79 ± 0.04^d^	76.27 ± 0.07^b^	70.66 ± 0.04^c^	76.45 ± 0.10^a^
TFC	78.17 ± 0.10^d^	99.08 ± 0.26^b^	81.04 ± 0.06^c^	113.30 ± 0.21^a^
TFLC	23.97 ± 0.03^d^	28.45 ± 0.04^b^	29.32 ± 0.03^a^	26.97 ± 0.05^c^
TAC	16.98 ± 1.74^d^	68.61 ± 1.65^a^	40.63 ± 1.74^b^	22.60 ± 0.48^c^
Proanthocyanidin	433.81 ± 1.11^d^	745.31 ± 2.90^a^	563.57 ± 3.32^b^	468.75 ± 0.00^c^
β‐glucan	11.93 ± 0.02^d^	16.20 ± 0.02^c^	16.43 ± 0.03^b^	19.57 ± 0.05^a^
β‐carotene	0.12 ± 0.02^d^	1.64 ± 0.01^b^	1.22 ± 0.02^c^	2.15 ± 0.02^a^
Alcohol by volume				
ABV (%)	–	1.09 ± 0.00^a^	0.73 ± 0.15^b^	1.00 ± 0.16^a^
Antioxidant properties				
DPPH (%)	84.51 ± 0.72^c^	93.79 ± 0.06^a^	94.13 ± 0.05^a^	91.42 ± 0.12^b^
ABTS (%)	88.28 ± 0.10^d^	96.19 ± 0.09^b^	97.69 ± 0.09^a^	94.87 ± 0.15^c^
FRAP (mM)	5.52 ± 0.01^d^	7.39 ± 0.02^a^	7.20 ± 0.02^b^	6.80 ± 0.01^c^

*Note*: Control represents unfermented RASB, RASB‐Y represents RASB inoculated with *Saccharomyces cerevisiae*, RASB‐LP represents RASB inoculated with *Lactobacillus plantarum* LP28, RASB‐LPY represents RASB inoculated with *Lactobacillus plantarum* LP28 and *Saccharomyces cerevisiae*. RASB represents rice, apple pomace, and sea buckthorn beverage. Means with different letters are significantly different (*p* < .05).

### 3.3 Effect of fermentation on β‐glucan and β‐carotene contents

Table 2 presents the β‐glucan and β‐carotene contents of RASB samples. The β‐glucan and β‐carotene contents in the fermented samples were significantly higher than that in the control. Notably, a 1.64‐fold and 17.92‐fold increase in β‐glucan and β‐carotene contents, respectively, was observed in RASB‐LPY compared to the control. This increase can be attributed to the enzymatic activity of the fermenting organism, leading to the breakdown of the cell wall and more efficient extractability of these compounds (Ogrodowczyk & Drabińska, 2021). Both β‐glucan and β‐carotene have been reported to exhibit several biological functions including, antidiabetic, antioxidative, and anti‐inflammatory properties (Guo et al., 2023; Rohmah et al., 2022). A strong positive correlation was observed between these compounds and antioxidant properties measured by DPPH, ABTS, and FRAP, as shown in Table S1. Similarly, pH was negatively correlated to β‐glucan and β‐carotene contents.

### 3.4 Effect of fermentation on the phytochemical contents of RASB

TPC, TFC, TFLC, and TAC of fermented RASB significantly increased compared to the control, as depicted in Table 2. The TPC and TFC values tend to be higher in the samples containing *Saccharomyces cerevisiae* (RASB‐Y and RASB‐LPY). However, RASB‐LPY increased by 42.13% and 44.94% for TPC and TFC compared to the control. In contrast, an increase of 22.32% was observed in RASB‐LP for TFLC as compared to the control. Noteworthy, fermentation enhanced the TAC and proanthocyanidin contents. However, the values were higher in the monoculture samples compared to the coculture, with the sample fermented with RASB‐Y exhibiting the highest values for TAC (68.61 ± 1.65 mg/100 g) and proanthocyanidin (745.31 ± 2.90 mg/100 g). The variation in phytochemical content in the fermented samples can be attributed to differences in the metabolic and enzymatic activity of the microorganisms. Furthermore, the lower TAC and proanthocyanidin levels observed in the coculture sample (RASB‐LPY) may be linked to interactions and competition between the microorganisms, which could impact the stability and release of these compounds (Boasiako et al., 2024). TPC was found to be positively correlated to TFC, TFLC, TAC, and proanthocyanidin. A strong positive correlation was also observed between TAC, TFLC, and proanthocyanidin. The increase in TPC and TFC can be attributed to the structural breakdown of cell walls and the hydrolytic enzymatic activities of the microorganisms. *Saccharomyces cerevisiae* and *L. plantarum* secret enzymes such as glucosidase, amylase, cellulose, and esterase that play significant roles in the structural breakdown of cell walls, thus leading to the extraction of phenolic compounds (Kwaw, Ma, Tchabo, Sackle, et al., 2018; Malini et al., 2024). More so, the increase in TPC and TFC can be attributed to the conversion of complex phenolic compounds into simpler forms. The TPC obtained in this study was lower than the TFC and proanthocyanidin, which might be linked to diverse extraction methods influencing bound phenolic compounds. These findings corroborate the report by Boasiako et al. (2024).

### 3.5 Antioxidative properties of fermented RASB

The antioxidant properties of food contribute to its health‐promoting benefits (Wang et al., 2022). The antioxidant capacity was measured by DPPH, ABTS, and FRAP assay, as detailed in Table 2. The fermented samples exhibited higher antioxidant properties as compared to the control. Specifically, the value ranged from 84.51 ± 0.72 (control) to 94.13 ± 0.05% (RASB‐LP), 88.28 ± 0.10 (control) to 97.69 ± 0.09% (RASB‐LP), and 5.52 ± 0.01 (control) to 7.39 ± 0.02 mM (RASB‐Y) for DPPH, ABTS, and FRAP, respectively. The antioxidative capacity of RASB measured by these assays demonstrates a strong positive correlation with TPC, TFLC, TAC, proanthocyanidin, β‐glucan, and β‐carotene. However, there was no observed correlation between the antioxidant properties and TFC. Furthermore, pH reduction, increased TTA, and total acid (TA, measured by HS‐SPME/GC–MS) play a significant role in the elevated bioactive content and antioxidant properties of RASB. These findings are in line with previous research by Kwaw, Ma, Tchabo, Apaliya, et al. (2018), highlighting the impact of fermentation on the functional properties of foods.

### 3.6 FTIR spectroscopy

FTIR spectroscopy, a technique based on the vibrational response of functional groups to infrared exposure, provides insight into the molecular structure of a wide range of compounds (Boasiako et al., 2024). Absorption peak 563.99 cm^−1^ was absent in the fermented samples but found in the control. The absence of this peak in the fermented might be due to its conversion to other compounds. Peak region 986.99 was observed in the control and the value tends to increase in RASB‐Y and RASB‐LP. However, this peak was not detected in RASB‐LPY. Interestingly, peak absorption at 1019.10, 1249.64, and 1715.92 cm^−1^ was unique to RASB‐LPY only. The spectra 500–1500 cm^−1^ are regarded as the fingerprint region and are characterized by the presence of sugars, organic acids, and alcohols. Most peaks observed in this region were higher in the fermented samples, except for the peak at 572.89 cm^−1^, where the control showed higher intensity than both RASB‐Y and RASB‐LP. Similarly, the peak at 864.66 cm^−1^ in the control surpassed that of RASB‐LP. The vibrational band in the region 1600–1700 cm^−1^ is related to C═O stretching and was found to increase in the fermented samples compared to the control. A vibration with a peak at 2923.53 cm^−1^ was detected in the control. The intensity increased in samples inoculated with *Saccharomyces cerevisiae* (RASB‐Y at 2925.3 cm^−1^ and RASB‐LPY at 2924.08 cm^−1^), while a decrease in intensity was observed in RASB‐LP (2923.51 cm^−1^). These values fall within the range reported by Budziak‐wieczorek et al. (2023) as being related to the stretching vibration of O–H bonds of carboxylic acid. Peak absorption at 3289.65 was noticed in the control, with decreasing intensity observed in the fermented samples, as depicted in Figure 3a. This finding confirms Ajayi et al.'s (2019) findings. The authors attributed the decrease in intensity to the dilution of the crystalline (amylose) region, resulting in the breakdown of glycosidic bonds. The control and RASB‐LPY had peaks of 3734.67 and 3734.68 cm^−1^, respectively, which was not detected in other samples. Budziak‐wieczorek et al. (2023) stated that a vibrational band in the range of 3000–3800 cm^−1^ is associated with the hydroxyl group (O–H stretch) present in alcohol, water, and phenol.

### 3.7 Sensory analysis using E‐nose

**FIGURE 1:**
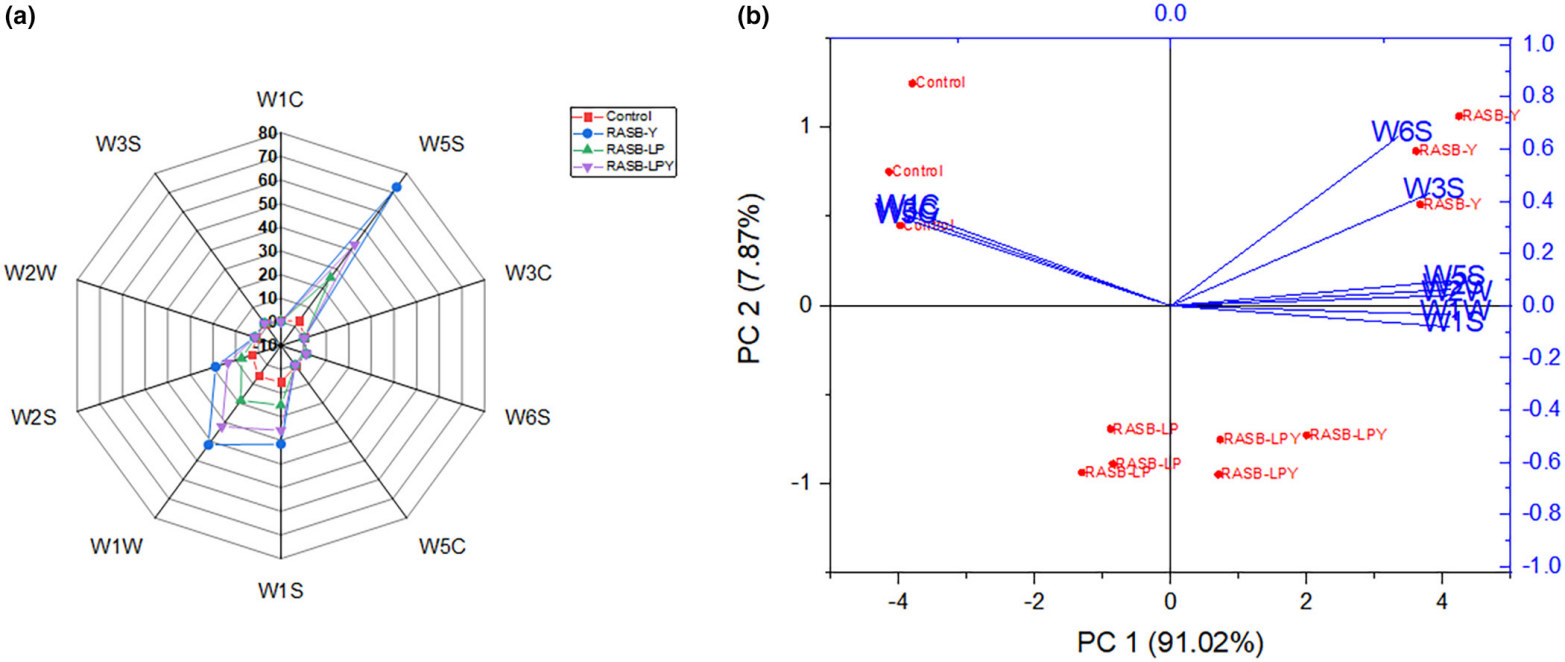
Radar plot (a) and principal component analysis (b) of e‐nose analysis of rice, apple pomace, and sea buckthorn beverage. Control represents unfermented RASB, RASB‐Y represents RASB inoculated with *Saccharomyces cerevisiae*, RASB‐LP represents RASB inoculated with *Lactobacillus plantarum* LP28, RASB‐LPY represents RASB inoculated with *Lactobacillus plantarum* LP28 and *Saccharomyces cerevisiae*. RASB represents rice, apple pomace, and sea buckthorn beverage.

This analysis demonstrates the capability of e‐nose to reflect the total volatile compounds present in the fermented samples. The results clearly showed that fermentation influences the sensory properties of RASB. The radar chart in Figure 1a illustrates the sensitivity of sensor discrimination in distinguishing between unfermented and fermented samples. A notable difference was observed in the response values of each sample. The highest values were recorded for sensors W5S (response to a broad range), W1S (broad range – methane), W1W (organic Sulfur compounds and terpenes), and W2S (broad range for alcohols). These values were significantly higher in the fermented samples, particularly in RASB‐Y, compared to the control. Among the fermented samples, RASB‐LP had the lowest value for W5S, W1S, W1W, and W2S. The sensitivity of the sensors indicates chemical modification caused by the metabolic activities of the fermenting microorganisms. Taiye and Zhou (2021) had earlier mentioned that sensor sensitivity can be attributed to chemical modification during pretreatment. Interestingly, the response values for sensors W1C (aromatic), W3C (aromatic), and W5C (aromatic–aliphatic) were higher in the control compared to the fermented samples. This might be attributed to high esters recorded in the control.

PCA analysis was employed to further elucidate the differences between the samples. PC1 accounted for 91.02% and PC2 for 7.87% of the variance, together explaining 98.89% of the total sample variation (Figure 1b). PC1 was primarily characterized by W1S, W1W, W2S, W2W, and W5S, whereas PC2 was associated with W1C, W3C, W6S (hydrogen), W5C, and W3S (methane–aliphatic). The control sample was mainly associated with W1C, W3C, and W5C, while RASB‐Y could be distinctly identified by W6S and W3S. RASB‐LPY and RASB‐Y exhibit a closer similarity, particularly in relation to W1W, W1S, W2W, W5S, and W2S as compared to RASB‐LP (Figure 1b). This finding aligns with the results of volatile compounds analysis.

### 3.8 Sensory evaluation

**FIGURE 2:**
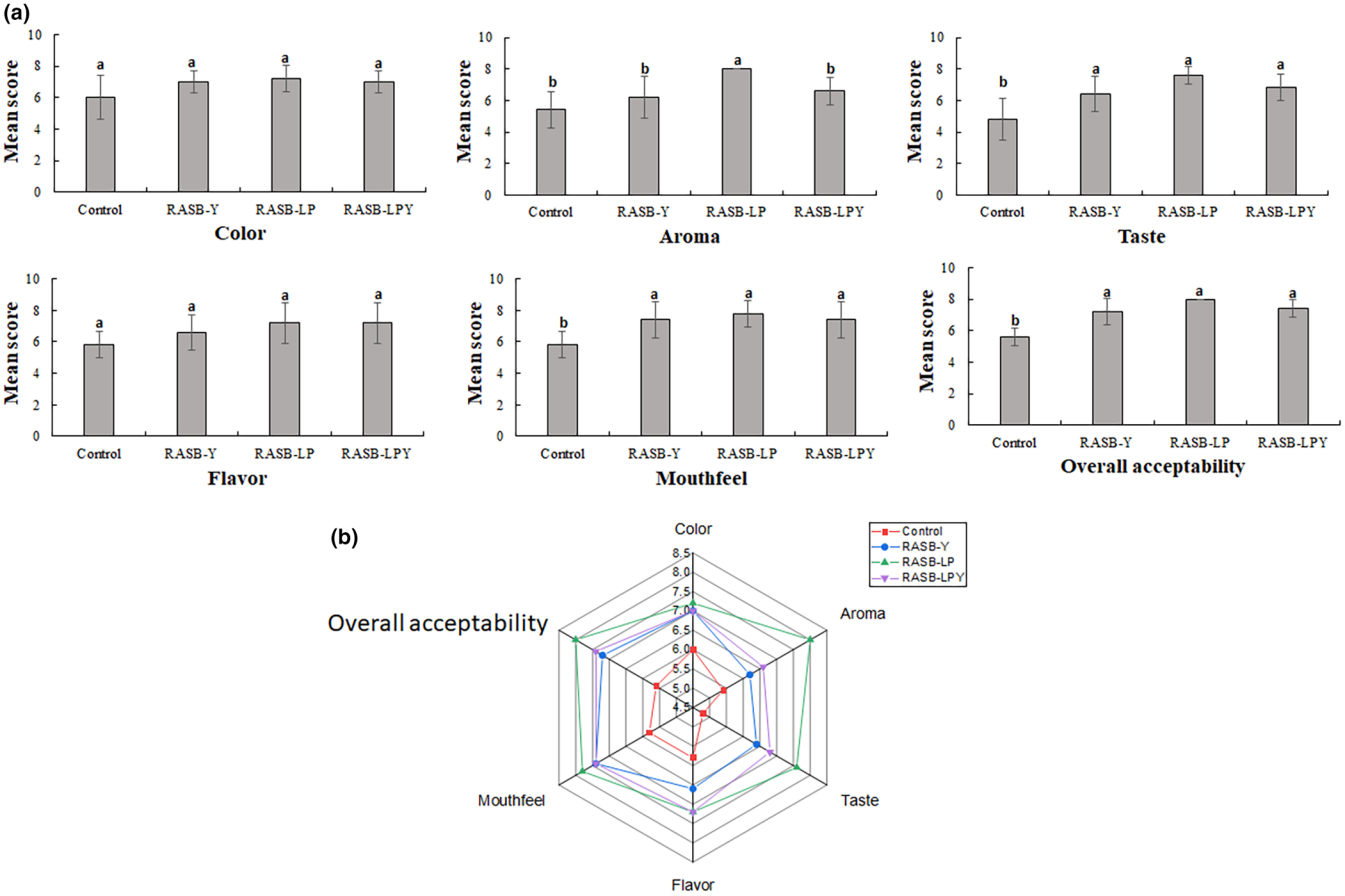
Mean score (a) and Radar plot (b) of sensory parameters of rice, apple pomace, and sea buckthorn beverage. Control represents unfermented RASB, RASB‐Y represents RASB inoculated with *Saccharomyces cerevisiae*, RASB‐LP represents RASB inoculated with *Lactobacillus plantarum* LP28, RASB‐LPY represents RASB inoculated with *Lactobacillus plantarum* LP28 and *Saccharomyces cerevisiae*. RASB represents rice, apple pomace, and sea buckthorn beverage. Means with different letters are significantly different (*p* < 0.05).

Sensory analysis was conducted using a 9‐point hedonic scale, focusing on parameters such as color, aroma, taste, flavor, mouthfeel, and overall acceptability, as illustrated in Figure 2A,B. When comparing the RASB samples, no significant difference was observed in color and flavor. In contrast, notable differences were evident in aroma, taste, mouthfeel, and overall acceptability between fermented and unfermented samples, although the fermented samples did not differ significantly from each other in these aspects except aroma where RASB‐LP was significantly higher. The fermented samples were most preferred with the highest overall acceptability recorded in RASB‐LP. The high mean scores for aroma, mouthfeel, and overall acceptability in RASB‐LP might be due to the presence and concentration of ethyl 8‐methylnonanoate, phenylmethyl pentanoate, and ethyl 2‐hydroxypropanoate (ethyl lactate). Ethyl 2‐hydroxypropanoate is known for contributing a mildly sweet, fruity, and creamy aroma to fermented food (Zhang et al., 2023).

### 3.9 Profiling of volatile compound using HS‐SPME/GC–MS

**FIGURE 3:**
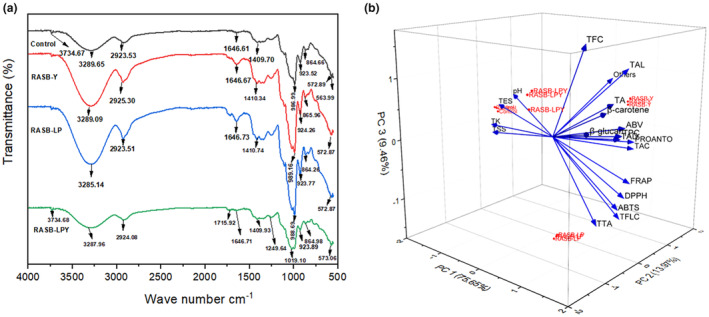
Fourier‐transform infrared spectroscopy (a) and 3D plot of rice, apple pomace, and sea buckthorn beverage (b). Control represents unfermented RASB, RASB‐Y represents RASB inoculated with *Saccharomyces cerevisiae*, RASB‐LP represents RASB inoculated with *Lactobacillus plantarum* LP28, RASB‐LPY represents RASB inoculated with *Lactobacillus plantarum* LP28 and *Saccharomyces cerevisiae*. RASB represents rice, apple pomace, and sea buckthorn beverage. ABV, alcohol by volume; Others, Oxime‐, methoxy‐phenyl, and 4‐octenoic acid, ethyl ether; PROANTO, proanthocyanidin; TA, total acid; TAC, total anthocyanin; TAD, total aldehyde; TAL, total alcohol; TES, total esters; TFC, total flavonoid content; TFLC, total flavonol content; TK, total ketones; TPC, total polyphenol content; TSS, total soluble solids; TTA, total titratable acidity.

A total of 49 volatile compounds, including 13 alcohols, 12 acids, 17 esters, 3 ketones, 2 aldehydes, and 2 other types, were identified and quantified, as shown in Table 3. The chromatogram is presented in Figure S1. A remarkable difference was observed in the total concentration of the identified compounds across the samples: 74.70 ± 2.05 ng/g in the control, 178.85 ± 1.51 ng/g in RASB‐Y, 84.32 ± 1.58 ng/g in RASB‐LP, and 144.60 ± 2.44 ng/g in RASB‐LPY. Esters contributed to 47.95% of the volatile compounds in the control, while alcohols accounted for 70.51%, 52.60%, and 63.06% of the identified volatile compounds in RASB‐Y, RASB‐LP, and RASB‐LPY, respectively. The high percentage of alcohols in RASB‐Y and RASB‐LPY samples is in line with the ABV, and e‐nose results (high value of sensor W2S related to broad alcohol) (Figure 1a). Higher alcohol content in fermented foods has been associated with the presence of *Saccharomyces cerevisiae*, as mentioned by Wu et al. (2022). 1‐Hexanol (8.36 ± 0.01 ng/g) and 2,3‐butanediol (8.90 ± 0.13 ng/g) occurred in higher concentrations in the control compared to the fermented samples, indicating that both compounds are indigenous to the raw materials. However, 2,3‐butanediol was not detected in RASB‐LP. 3‐Methyl‐1‐butanol and phenylethyl alcohol were the predominant alcohols in the fermented samples and were not detected in the control sample. These compounds were significantly higher in samples containing *Saccharomyces cerevisiae*, particularly RASB‐Y. Specifically, there was a 2.77‐fold increase in 3‐methyl‐1‐butanol and a 4.44‐fold increase in phenylethyl alcohol in RASB‐Y compared to RASB‐LP. Similarly, Yan et al. (2022) have reported 3‐methyl‐1‐butanol and phenylethyl alcohol as major alcohols in a fermented traditional Chinese beverage. 3‐methyl‐1‐butanol, which is derived from leucine, is characterized by a strong fruity aroma, while phenylethyl alcohol is known to impart a rose‐like floral odor (Chen et al., 2022; Kaprasob et al., 2018). 3‐methyl‐1‐butanol plays a significant role in the aromatic characteristics of various beverages, including wines and beers (Giri et al., 2018). The elevated concentration of 3‐methyl‐1‐butanol and 3‐methyl‐butanoic acid in the fermented samples might be attributed to the hydrolysis of 3‐methylbutyl 3‐methylbutanoate, potentially explaining the lower concentration of this ester in these samples. Previous research by Liang et al. (2022) associated ester reduction during juice fermentation with the volatility of esters as well as the high esterase activity of the fermenting microorganisms. 2‐(methylthio) ethanol was detected only in RASB‐Y, while 2‐methyl‐1‐pentanol occurred in RASB‐LP only and 1‐dodecanol was detected only in RASB‐LPY. Diverse acids were found in the fermented samples, while only a few were present in the control and at low concentrations. The predominant acid in RASB samples was 3‐methyl‐butanoic acid, with concentrations ranging from 4.55 ± 0.18 (control) to 8.80 ± 0.18 ng/g (RASB‐LP). Acetic acid was observed at higher concentrations in samples containing *Saccharomyces cerevisiae*, with the highest concentration recorded in RASB‐Y (6.25 ± 0.06 ng/g). Dodecanoic acid and 2‐methyl‐propanoic acid were only present in samples inoculated with *Saccharomyces cerevisiae*. On the other hand, benzoic acid was discovered only in samples inoculated with *L*. *plantarum* (RASB‐LP and RASB‐LPY). Benzoic acid has been reported to be associated with fermented foods and it acts as a preservative against spoilage microorganisms (Yerlikaya et al., 2021). RASB‐LPY exhibited a broader range of acids, unique to the sample was the presence of L‐lactic acid, hexadecanoic acid, and tetradecanoic acid, which were not detected in either the control or other fermented samples. This might explain the absorption peaks peculiar to this sample in Figure 3a. More esters were present in the control compared to the fermented samples, with RASB‐LPY having the least both in number and concentrations. Esters such as 3‐methyl‐1‐butanol benzoate, ethyl 3‐hydroxy‐3‐methylbutanoate, isobutyl nonyl oxalate, and 1‐methylpropylpentanoate were unique to the control sample. In contrast, 2‐phenylethyl acetate and ethyl decanoate were only found in RASB‐Y, while phenylmethyl pentanoate was observed solely in RASB‐LP. Ethyl decanoate, hexyl salicylate, and methyl, 2‐hydroxy propanoate were distinct to RASB‐LPY only. The predominant esters were 3‐methylbutyl 3‐methylbutanoate, with the control exhibiting the highest concentration (15.39 ± 0.32 ng/g), while RASB‐LP (7.18 ± 0.21 ng/g) recorded the highest concentration among the fermented samples. Ethyl 8‐methylnonanoate was found only in the control and RASB‐LP, while Ethyl 2‐hydroxypropanoate was detected only in samples containing *L. plantarum* (RASB‐LP and RASB‐LPY). Acetoin emerged as the predominant ketone d in RASB samples, with its levels varying from 0.58 ± 0.03 ng/g (RASB‐Y) to 5.37 ± 0.19 ng/g (control). 6‐Methyl 5‐hepten‐2‐one was present in all samples except RASB‐LP, showing the highest concentration in the control. In contrast, 2‐heptanone appeared exclusively in the control and RASB‐LP, with the latter recording the peak concentration (0.78 ± 0.03 ng/g). In the case of aldehydes, these compounds are found solely in the fermented samples. Specifically, benzaldehyde was unique to RASB‐Y, whereas 2,4‐dimethylbenzaldehyde was prevalent in all the fermented samples. The absence of benzaldehyde in the samples inoculated with *L. plantarum* (RASB‐LP and RASB‐LPY) might be linked to its oxidation to benzoic acid, thereby accounting for the presence of benzoic acid only in these samples. This observation aligns with the findings of Yerlikaya et al. (2021), who reported that LAB can convert benzaldehyde into benzoic acid through oxidation. The compound oxime‐, methoxy‐phenyl was identified solely in RASB‐Y at concentration 3.68 ± 0.20 ng/g, and 4‐octenoic acid, ethyl ether was only detected in the control. The dendrogram of volatile compounds (Figure S2) displays close a similarity between RASB‐Y and RASB‐LPY. RASB‐LP is closer to the RASB‐Y and RASB‐LPY clusters, while the control is farther from these fermented samples.

**TABLE 3:** Concentration of volatile compounds identified in fermented RASB samples by HS‐SPME‐GC/MS.

Volatiles	S/N	Compound name	Concentration (ng/g)				
			Control	RASB‐Y	RASB‐LP	RASB‐LPY	CAS
Alcohols							
	AL1	1‐Butanol	0.36 ± 0.00^a^	nd	nd	nd	71‐36‐3
	AL2	1‐Hexanol	8.36 ± 0.01^a^	2.52 ± 0.03^c^	1.58 ± 0.01^d^	4.02 ± 0.10^b^	111‐27‐3
	AL3	1‐Octen‐3‐ol	0.96 ± 0.01^a^	0.49 ± 0.01^c^	0.65 ± 0.02^b^	nd	3391‐86‐4
	AL4	1‐Pentanol	0.96 ± 0.02^a^	nd	nd	nd	71‐41‐0
	AL5	2,3‐Butanediol	8.90 ± 0.13^a^	2.86 ± 0.02^b^	nd	2.41 ± 0.12^c^	513‐85‐9
	AL6	2,4‐Di‐tert‐butylphenol	0.15 ± 0.01^b^	nd	0.44 ± 0.03^a^	nd	96‐76‐4
	AL7	2‐Octanol	1.88 ± 0.02^b^	0.87 ± 0.02^d^	1.63 ± 0.01^c^	2.28 ± 0.11^a^	123‐96‐6
	AL8	2‐Octen‐1‐ol	0.40 ± 0.01^a^	nd	nd	nd	18409‐17‐1
	AL9	1‐Butanol, 3‐methyl‐	nd	85.04 ± 0.48^a^	30.68 ± 0.27^c^	57.29 ± 0.61^b^	123‐51‐3
	AL10	Ethanol, 2‐(methylthio)‐	nd	0.28 ± 0.01^a^	nd	nd	5271‐38‐5
	AL11	Phenylethyl Alcohol	nd	34.06 ± 0.30^a^	7.67 ± 0.19^c^	24.78 ± 0.53^b^	60‐12‐8
	AL12	1‐Pentanol, 2‐methyl‐	nd	nd	1.70 ± 0.04^a^	nd	105‐30‐6
	AL13	1‐Dodecanol	nd	nd	nd	0.41 ± 0.01^a^	112‐53‐8
Subtotal			21.97 ± 0.16^d^	126.11 ± 0.48^a^	44.35 ± 0.53^c^	91.18 ± 0.98^b^	
Acids							
	AC1	Acetic acid	2.73 ± 0.02^d^	6.25 ± 0.06^a^	2.92 ± 0.04^c^	4.06 ± 0.04^b^	64‐19‐7
	AC2	3‐methyl‐Butanoic acid	4.55 ± 0.18^d^	6.85 ± 0.07^c^	8.80 ± 0.18^a^	7.75 ± 0.25^b^	503‐74‐2
	AC3	Hexanoic acid	1.11 ± 0.12^b^	4.24 ± 0.20^a^	4.32 ± 0.22^a^	4.38 ± 0.24^a^	142‐62‐1
	AC4	Dodecanoic acid	nd	0.30 ± 0.02^b^	nd	0.40 ± 0.02^a^	143‐7‐7
	AC5	Heptanoic acid	nd	0.30 ± 0.04^a^	0.26 ± 0.01^ab^	0.23 ± 0.02^b^	111‐14‐8
	AC6	Decanoic acid	nd	0.289 ± 0.08^a^	2.47 ± 0.07^a^	4.40 ± 0.28^a^	334‐48‐5
	AC7	Octanoic acid	nd	8.78 ± 0.23^a^	4.30 ± 0.19^c^	7.28 ± 0.29^b^	124‐7‐2
	AC8	Propanoic acid, 2‐methyl‐	nd	3.24 ± 0.17^a^	nd	2.8 ± 0.12^b^	79‐31‐2
	AC9	Benzoic acid	nd	nd	0.29 ± 0.03^a^	0.25 ± 0.01^b^	65‐85‐0
	AC10	L‐Lactic acid	nd	nd	nd	3.44 ± 0.07^a^	79‐33‐4
	AC11	Hexadecanoic acid	nd	nd	nd	1.57 ± 0.10^a^	57‐10‐3
	AC12	Tetradecanoic acid	nd	nd	nd	0.41 ± 0.01^a^	544‐63‐8
Subtotal			8.40 ± 0.28^d^	32.85 ± 0.75^b^	23.36 ± 0.65^c^	36.98 ± 1.29^a^	
Esters							
	EST1	1‐Butanol, 3‐methyl‐, benzoate	4.56 ± 0.41^a^	nd	nd	nd	94‐46‐2
	EST2	8‐Methylnonanoic acid, ethyl ester	0.15 ± 0.01^b^	nd	0.49 ± 0.04^a^	nd	0‐0‐0
	EST3	Butanoic acid, 3‐methyl‐, 2‐phenylethyl ester	0.46 ± 0.05^a^	0.39 ± 0.02^b^	0.28 ± 0.01^c^	nd	140‐26‐1
	EST4	Butanoic acid, 3‐methyl‐, 3‐methylbutyl ester	15.39 ± 0.32^a^	4.75 ± 0.04^c^	7.18 ± 0.21^b^	4.89 ± 0.15^c^	659‐70‐1
	EST5	Ethyl 3‐hydroxy‐3‐methylbutanoate	0.43 ± 0.04^a^	nd	nd	nd	18267‐36‐2
	EST6	Hexanoic acid, ethyl ester	6.62 ± 0.09^a^	0.61 ± 0.05^c^	0.97 ± 0.05^b^	nd	123‐66‐0
	EST7	Isopentyl hexanoate	4.54 ± 0.34^a^	2.86 ± 0.07^c^	3.01 ± 0.03^c^	3.58 ± 0.15^b^	2198‐61‐0
	EST8	Octanoic acid, ethyl ester	2.78 ± 0.24^a^	2.44 ± 0.31^a^	0.18 ± 0.02^b^	nd	106‐32‐1
	EST9	Oxalic acid, isobutyl nonyl ester	0.46 ± 0.02^a^	nd	nd	nd	0‐0‐0
	EST10	Pentanoic acid 1‐methylpropyl ester	0.42 ± 0.02^a^	nd	nd	nd	23361‐74‐2
	EST11	Acetic acid, 2‐phenylethyl ester	nd	0.35 ± 0.05^a^	nd	nd	103‐45‐7
	EST12	Decanoic acid, ethyl ester	nd	1.96 ± 0.06^a^	nd	nd	110‐38‐3
	EST13	Pentanoic acid, phenylmethyl ester	nd	nd	0.24 ± 0.02^a^	nd	10361‐39‐4
	EST14	Propanoic acid, 2‐hydroxy‐, ethyl ester	nd	nd	0.55 ± 0.03^a^	0.32 ± 0.03^b^	97‐64‐3
	EST15	Decanoic acid, ethyl ester	nd	nd	nd	0.97 ± 0.02^a^	110‐38‐3
	EST16	Hexyl salicylate	nd	nd	nd	0.94 ± 0.02^a^	6259‐76‐3
	EST17	Propanoic acid, 2‐hydroxy‐, methyl ester	nd	nd	nd	1.36 ± 0.05^a^	2155‐30‐8
Subtotal			35.82 ± 1.38^a^	13.37 ± 0.56^b^	12.90 ± 0.39^b^	12.07 ± 0.38^b^	
Ketones							
	KET1	2‐Heptanone	0.19 ± 0.01^b^	nd	0.78 ± 0.03^a^	nd	110‐43‐0
	KET2	5‐Hepten‐2‐one, 6‐methyl‐	2.01 ± 0.04^a^	0.62 ± 0.03^c^	nd	0.73 ± 0.04^b^	110‐93‐0
	KET3	Acetoin	5.37 ± 0.19^a^	0.58 ± 0.03^c^	1.48 ± 0.03^b^	0.71 ± 0.02^c^	513‐86‐0
Subtotal			7.58 ± 0.24^a^	1.20 ± 0.05^d^	2.26 ± 0.05^b^	1.45 ± 0.06^c^	
Aldehydes							
	ALD1	Benzaldehyde	nd	0.81 ± 0.03^a^	nd	nd	100‐52‐7
	ALD2	Benzaldehyde, 2,4‐dimethyl‐	nd	1.04 ± 0.04^a^	1.45 ± 0.05^a^	1.92 ± 0.03^a^	15764‐16‐6
Subtotal			nd	1.85 ± 0.06^a^	1.45 ± 0.05^b^	1.92 ± 0.03^a^	
Others							
	OTHERS1	Oxime‐, methoxy‐phenyl‐	nd	3.68 ± 0.20^a^	nd	nd	64‐17‐5
	OTHERS2	4‐Octenoic acid, ethyl ether	0.94 ± 0.03^a^	nd	nd	nd	0‐0‐0
Subtotal			0.94 ± 0.03^b^	3.68 ± 0.20^a^			
Total volatiles			74.70 ± 2.05^d^	178.85 ± 1.51^a^	84.32 ± 1.58^c^	144.60 ± 2.44^b^	

*Note*: Control represents unfermented RASB, RASB‐Y represents RASB inoculated with *Saccharomyces cerevisiae*, RASB‐LP represents RASB inoculated with *Lactobacillus plantarum* LP28, RASB‐LPY represents RASB inoculated with *Lactobacillus plantarum* LP28 and *Saccharomyces cerevisiae*. RASB represents rice, apple pomace, and sea buckthorn beverage. Means with different letters are significantly different (*p* < .05).

### 3.10 Principal component analysis (PCA)

The PCA provides insight into the impact of fermenting microorganisms on RASB samples. It revealed that PC1 (75.65%), PC2 (13.97%), and PC3 (9.46%) account for 99.08% of the total variance, as illustrated in Figure 3b. The samples clustered into three distinct groups based on specific features. The control is closely related to elevated pH, TES (Total esters), TK (total ketones), and TSS (total soluble solids). In contrast, RASBY and RASB‐LPY were characterized by TPC, TFC, TAC, PROANTHO (proanthocyanidin), TA (total acid), TAL (total alcohol), TAD (total aldehyde), β‐carotene, β‐glucan, ABV, and others (oxime‐, methoxy‐phenyl). Meanwhile, RASB‐LP is associated with TTA, TFLC, DPPH, and ABTS. PC1 was mainly influenced by TPC, ABV, and TAD which best described samples inoculated with *Saccharomyces cerevisiae* (RASB‐Y and RASB‐LPY). TSS, TES, and TK effectively characterized the control. PC2 is primarily related to TAC and PROANTHO characterizing the monoculture samples (RASB‐Y and RASB‐LP), as well as oxime‐, methoxy‐phenyl, peculiar to RASB‐Y. Furthermore, PC3 comprises TFC, linked to RASB‐LPY.

## 4 CONCLUSION

This study demonstrated that fermentation with probiotic *Lactobacillus plantarum* LP28 and *Saccharomyces cerevisiae* significantly enhances the bioactive content, and functional and sensory properties of RASB. Specifically, fermentation notably improved TPC, TFC, β‐carotene, and β‐glucan levels, with coculture sample (RASB‐LPY) showing substantial increases. RASB‐LP containing *Lactobacillus plantarum* displayed superior TFLC level, antioxidant properties and was highly rated for its aroma and overall acceptability. Additionally, 3‐methyl‐1‐butanol, contributing a fruity and sweet aroma, was abundant in fermented samples, and benzoic acid was detected in *Lactobacillus plantarum* samples (RASB‐LP and RASB‐LPY), indicating natural preservation potential. FTIR analysis revealed significant changes in the structural properties of the RASB samples, with unique peaks in RASB‐LPY, while HS‐SPME/GC–MS identified a diverse range of organic acids in the same sample, underscoring the complexity of the fermentation process. These findings highlight the potential for optimizing fermentation conditions to further improve the quality of coculture fermented RASB. The development of such fermented beverages, incorporating fruit and pomace, not only promises beverages with enhanced quality but also offers a sustainable approach to mitigating waste and addressing environmental concerns. As a future research direction, we suggest conducting comprehensive shelf life studies to evaluate the storage stability, microbial safety, and changes in bioactive compounds and sensory properties of the beverage over time under various storage conditions.

## AUTHOR CONTRIBUTIONS

**Afusat Yinka Aregbe:** Conceptualization (equal); formal analysis (equal); investigation (equal); methodology (equal); writing – original draft (equal). **Turkson Antwi Boasiako:** Methodology (equal); writing – review and editing (equal). **YuQing Xiong:** Formal analysis (equal); investigation (equal). **Md. Hafizur Rahman:** Writing – review and editing (equal). **Yongkun Ma:** Conceptualization (equal); project administration (equal); supervision (equal).

## FUNDING INFORMATION

This research was funded by Zhenjiang Key Research and Development Program (Modern Agriculture) grant number NY2020020.

## CONFLICT OF INTEREST STATEMENT

The authors declare no conflicts of interest.

## ETHICS STATEMENT

No ethical approval is required for the experiment. Nevertheless, informed consent was obtained from the participant before the sensory test was carried out. Additionally, this article does not contain any studies involving animals conducted by any of the authors.

## Supporting information


Data S1.

## Data Availability

Data will be made available based on request.
