# Supplementary material for: Probiotic Lactobacillus plantarum LP28 and Saccharomyces cerevisiae improve the bioactive content and quality of fruit‐based rice beverage
Source: Food Sci Nutr. 2024 Oct 6;12(11):9340–52. doi: 10.1002/fsn3.4462 (PMC11606879; doi:10.1002/fsn3.4462)
Supplement: Supplementary file 1 — Data S1. [file FSN3-12-9340-s001.docx]

Table S1: Pearson correlation c0efficient of pH, TTA, TSS, phytochemicals, antioxidant properties and volatiles

|  | TPC | TFC | TFLC | TAC | PRO-  ANTHO | FRAP | DPPH | ABTS | ABV | β-glucan | β-  carotenene | PH | TTA | TSS | TAL | TA | TE | TK | TAD | Others |
| --- | --- | --- | --- | --- | --- | --- | --- | --- | --- | --- | --- | --- | --- | --- | --- | --- | --- | --- | --- | --- |
| TPC | 1 |  |  |  |  |  |  |  |  |  |  |  |  |  |  |  |  |  |  |  |
| TFC | 0.76** | 1 |  |  |  |  |  |  |  |  |  |  |  |  |  |  |  |  |  |  |
| TFLC | 0.80** | 0.22 | 1 |  |  |  |  |  |  |  |  |  |  |  |  |  |  |  |  |  |
| TAC | 0.58* | 0.13 | 0.70* | 1 |  |  |  |  |  |  |  |  |  |  |  |  |  |  |  |  |
| PRO-  ANTHO | 0.58* | 0.16 | 0.68* | 0.99** | 1 |  |  |  |  |  |  |  |  |  |  |  |  |  |  |  |
| FRAP | 0.91** | 0.43 | 0.96** | 0.78** | 0.77** | 1 |  |  |  |  |  |  |  |  |  |  |  |  |  |  |
| DPPH | 0.89** | 0.38 | 0.98** | 0.72** | 0.70* | 0.99** | 1 |  |  |  |  |  |  |  |  |  |  |  |  |  |
| ABTS | 0.87** | 0.35 | 0.99** | 0.64* | 0.62* | 0.97** | 0.99** | 1 |  |  |  |  |  |  |  |  |  |  |  |  |
| ABV | 0.97** | 0.74** | 0.77** | 0.62* | 0.63* | 0.90** | 0.86** | 0.83** | 1 |  |  |  |  |  |  |  |  |  |  |  |
| B-  GLUCAN | 0.90** | 0.83** | 0.61* | 0.17 | 0.17 | 0.70* | 0.71* | 0.73** | 0.84** | 1 |  |  |  |  |  |  |  |  |  |  |
| B-  CAAROTENE | 0.96** | 0.89** | 0.64* | 0.37 | 0.37 | 0.77** | 0.75** | 0.74** | .93** | 0.97** | 1 |  |  |  |  |  |  |  |  |  |
| pH | -0.91** | -0.59* | -.845** | -0.35 | -0.34 | -.857** | -0.89** | -0.92** | -0.85** | -0.93** | -0.89** | 1 |  |  |  |  |  |  |  |  |
| TTA | 0.77** | 0.32 | 0.89** | 0.38 | 0.35 | 0.84** | 0.88** | 0.93** | 0.73** | 0.75** | 0.70* | -0.92** | 1 |  |  |  |  |  |  |  |
| TSS | -0.95** | -0.68* | -0.80** | -0.61* | -0.62* | -0.90** | -0.88** | -0.86** | -0.99** | -0.83** | -0.90** | 0.86** | -0.77** | 1 |  |  |  |  |  |  |
| TAL | 0.84** | 0.77** | 0.50 | 0.72** | 0.74** | 0.72** | 0.63* | 0.55 | 0.86** | 0.62* | 0.79** | -0.54 | 0.35 | -0.81** | 1 |  |  |  |  |  |
| TA | 0.97** | 0.89** | 0.64* | 0.45 | 0.46 | 0.79** | 0.76** | 0.74** | 0.94** | 0.93** | .99** | -0.85** | 0.65* | -0.91** | 0.86** | 1 |  |  |  |  |
| TES | -0.97** | -0.62* | -0.89** | -0.54 | -0.53 | -0.94** | -0.95** | -0.95** | -0.93** | -0.89** | -0.91** | 0.97** | -0.90** | 0.93** | -0.69* | -0.90** | 1 |  |  |  |
| TK | -0.99** | -0.68* | -0.86** | -0.64* | -0.63* | -0.95** | -0.93** | -0.91** | -0.97** | -0.87** | -0.93** | 0.917** | -0.81** | 0.95** | -0.81** | -0.94** | 0.98** | 1 |  |  |
| TAD | 0.99** | 0.76** | 0.80** | 0.56 | 0.56 | 0.90** | 0.89** | 0.87** | 0.97** | 0.91** | 0.97** | -0.92** | 0.78** | -0.95** | 0.82** | 0.97** | -0.97** | -0.99** | 1 |  |
| Others | 0.20 | 0.10 | 0.14 | 0.79** | 0.82** | 0.29 | 0.19 | 0.07 | 0.29 | -0.20 | 0.05 | 0.18 | -0.21 | -0.24 | 0.64* | 0.17 | -0.04 | -0.22 | 0.17 | 1 |

** Correlation is significant at the 0.01 level (2-tailed).

* Correlation is significant at the 0.05 level (2-tailed).

TPC stands for total polyphenol content, TFC stands for total flavonoid content, TFLC stands for total flavonol content, TAC stands for total anthocyanin, PROANTO stands for proanthocyanidin, ABV stands for alcohol by volume, TTA stands for total titratable acidity, TSS stands for total soluble solids, TAL stands for total alcohol, TA stands for total acid, TES stands for total esters, TK stands for total ketones, TAD stands for total aldehyde, Others stand for Oxime-, methoxy-phenyl-, and 4-Octenoic acid, ethyl ether


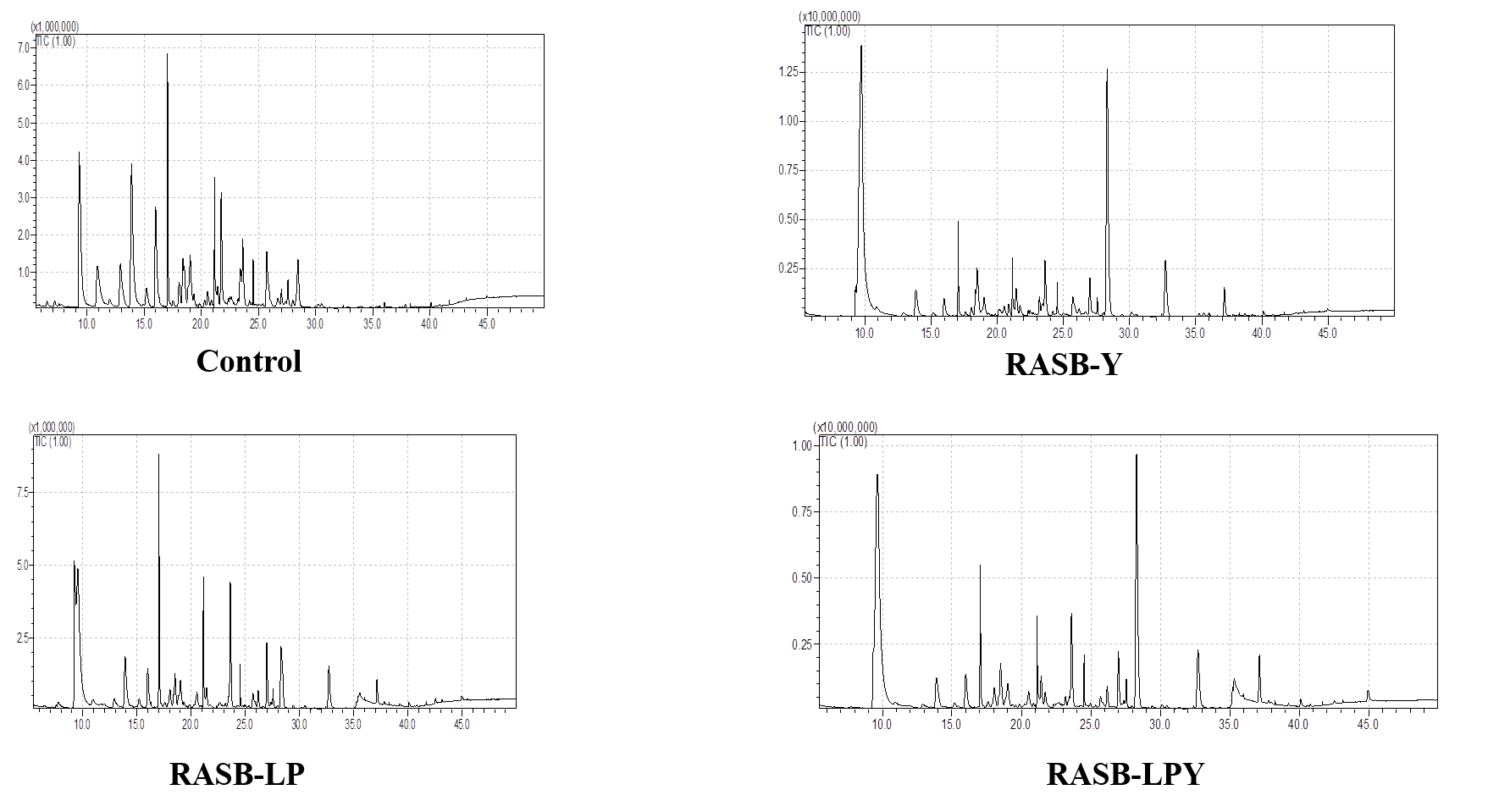


Fig.S1


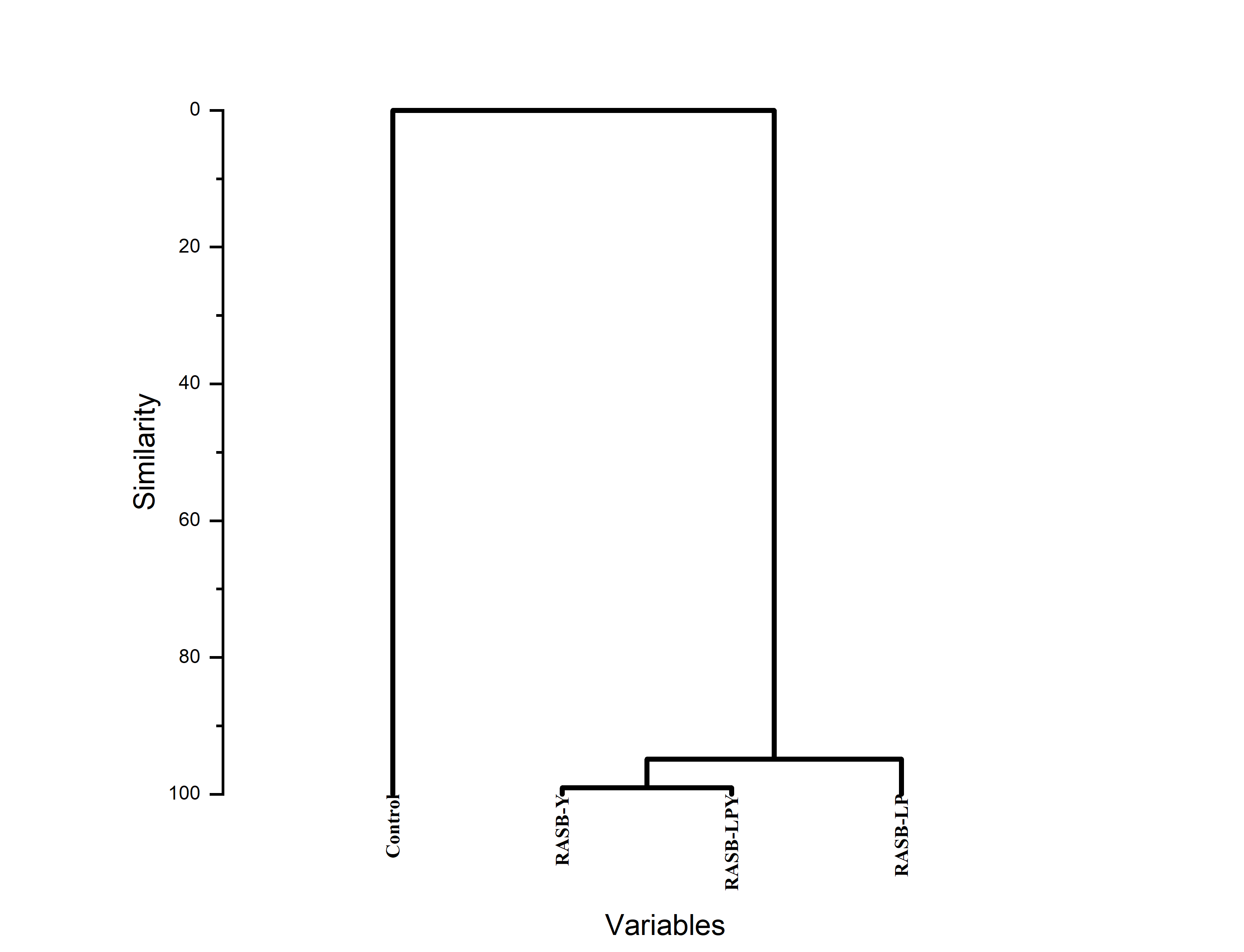


Fig. S2
